# 

**Published:** 1914-08-22

**Authors:** 


### CONTENTS.

PAGE

**The Hospital Necessities of the Civil Population** ... ... ... ... ... ... ... 561

**Some Non-Combatant Responsibilities** ... 562

**Hospital and Institutional News** ... ... ... 563

The Hospital's Special Correspondents — A London Hospital Secretary Leaves for Active Service—How Ex-Hospital Men may Help—The Position at the German Hospital—Poor-Law Infirmaries and Food Supplies: Asylums Board's Decision—A Board Room as a Children's Ward—Suggested Diversion of Surplus Panel Funds—The Pivot of the Voluntary Hospital—A Warning of the Need for Public Support—The Bathing of Refractory Infirmary Patients—Poor-Law Institutions and the War —Poor-Law Infirmaries and their Contractors—Hospitals and their Food Contracts —The Croix Rouge in Brussels—The Use of Chlorine in Hospital Laundries—A London Hospital President's Example—Anti-typhoid Inoculation and the Army—The War Office and Nursing Homes—The Abuse of the Red Cross—Poor-Law Officers as Volunteers—New Secretary of Manchester Children's Hospital—From Private House to Hospital: A Mistaken Policy—The Offer of the French Hospital—The Use and Abuse of Ambulance Ships—Co-operation of the Western Hospitals—Caterpillars and Bubonic Plague—The Kerby Private Wards at Newbury—The Drug Market and Panic Ordering—To our Readers.

PAGE

**A STATE OF WAR.**

**The Country and the Care of the Sick and Wounded** ... ... ... ... ... ... 569

Some Dangers and Mistakes to be Avoided.

Position of the Voluntary Hospitals.

The Problem before the London Hospitals.

Territorial Hospitals and Nursing Service.

[See over.]

### CONTENTS—(*continued*).

PAGE

**Hospitals and the War** ... ... ... ... 575

Grateful Thanks to our War Correspondents.

The London Hospital's Arrangements: Guy's Hospital—Metropolitan Hospital—St. George's Hospital—St. Mary's Hospital—Westminster Hospital—University College Hospital.

Provincial Hospital Arrangements: Barnsley—Bath—Barnstaple—A Southern Territorial Hospital—Bristol—Cambridge—Essex—Hospital Co-operation in Exeter—Gravesend—Guildford—Ipswich—Lancashire—Leicester—Lincolnshire—Middlesbrough—Oldham—Portsmouth—Hartshill, Staffordshire—Stalybridge—Torquay—Tunbridge Wells—Wakefield—Walsall (Staffs)—Yarmouth.

PAGE

**The Institutional Worker** ... ... (Suppl.) 1

How to Start a Hospital Laundry.

Questions for August and September.

Questions Invited.

The Sick and in Need.

Employment and Training.

Practical Matters.

Miscellaneous.

Acknowledgments.

**The Editor's Letter-Box** ... ... (Suppl.) 3

The War and Poor-Law Infirmaries—A Post No One is Asked to Fill—Hospital Drug Supply and the War.

**The Leicester Hospital Saturday Society** (Suppl.) 3

**Institutional Needs** ... ... ... (Suppl.) 3

